# Generation of a syngeneic orthotopic transplant model of prostate cancer metastasis

**DOI:** 10.18632/oncoscience.88

**Published:** 2014-10-15

**Authors:** Leigh Ellis, Kristin Lehet, ShengYu Ku, Gissou Azabdaftari, Roberto Pili

**Affiliations:** ^1^ Genitourinary Program, Roswell Park Cancer Institute, Buffalo NY, USA; ^2^ Department of Pharmacology and Therapeutics, Roswell Park Cancer Institute, Buffalo NY, USA; ^3^ Department of Cancer Prevention and Pathology, Roswell Park Cancer Institute, Buffalo NY, USA; ^4^ Department of Pathology, Roswell Park Cancer Institute, Buffalo NY, USA; ^5^ Department of Medicine, Roswell Park Cancer Institute, Buffalo NY, USA

**Keywords:** metastasis, prostate cancer, MYC, mouse models, castrate resistant prostate cancer

## Abstract

Progression to metastatic disease is the primary cause of mortality in men with prostate cancer (PCa). Mouse models which progress with spontaneous metastasis are limited. Such models would allow for extensive studies of molecular mechanisms of metastasis, and more definite pre- clinical therapy trials. Orthotopic murine models have been described; however a limiting biology of these models is their lack of an intact immune system. Within, we describe the development of an androgen sensitive and castrate resistant tractable orthotopic murine syngeneic (immune competent) model of prostate cancer. Both models develop primary tumors which spontaneously progress to metastatic disease in lymph tissue. These models will allow for more complete mechanistic and therapeutic studies in a short time period.

## INTRODUCTION

Prostate cancer (PCa) is among the top three most prevalent cancers among males [1] and the second leading cause of cancer related deaths in men in the United States [2]. An estimated 233,000 new prostate cancer patients were diagnosed in 2014 with an estimated total of 29,480 deaths due to prostate cancer [2].

Recent advances of FDA approved therapies including Enzalutamide (MDV3100), Zytiga (Abiraterone Acetate) and Radium-223 have displayed potential in the clinic. Unfortunately, these new therapies only offer modest survival in patients with mCRPC, and novel therapies are still urgently required which achieve sustainable suppression of tumor growth.

Mouse models are a powerful tool to conduct preclinical or ‘proof of concept’ therapeutic experiments to expedite results to clinical trial. Death from PCa does not occur because of formation of the primary tumor, but rather the progression to metastatic disease. This highlights the primary concern for PCa patients is the successful treatment of metastatic tumors. a recent meeting held by the Prostate Cancer Foundation which addressed issues hampering the translation of bench to bedside discoveries resulting from research generated in animal models [3]. Concerns raised from this meeting included a lack of understanding of the molecular events that define tumorigensis and a lack of tools for studying tumor-host interactions [3].

Current transgenic mouse models of PCa very rarely reflect molecular features of prostate cancer initiation and progression that correlate with human disease. Also, development of castrate resistant or metastatic disease is limited. Experimental mouse models of metastatic PCa involve tail-vein or intracardiac injections, which generates an artificial metastatic environment. Further, these models do not accurately capture true progression of metastatic PCa, as tumors have not undergone a natural ‘metastatic evolution’ process, which includes escape of the primary tumor and microenvironment and ability to nest and grow at distal sites. Also, experimental models of metastatic PCa often involve human cell lines or tissue, meaning there is not a true representation of the immune system, which is a critical component of the therapeutic environment and metastatic niche. These are significant limiting factors for preclinical animal models, and represent an urgent need to develop models which capture the full metastatic cascade; from primary tumor to development of metastatic disease, in the presence of an intact immune system. Models which reflect human PCa and allow for assessment of primary and metastatic tumors in the presence of an intact immune system would allow for more in depth understanding of molecular switches which drive metastatic disease, and identify more reliable therapeutic targets. One recent transgenic model that shares molecular features with human prostate cancer expresses c-Myc exclusively within the luminal epithelium cells of the prostate [4]. This mouse model develops organ confined invasive adenocarcinoma, without progression to castrate resistant or metastatic disease. Further, an androgen sensitive cell line (Myc-CaP/AS) was derived from this model [5], allowing for the generation of a syngeneic transplant mouse model of PCa.

## RESULTS AND DISCUSSION

We had previously demonstrated the development of a subcutaneous castrate resistant tumor [6] derived from an androgen sensitive subcutaneous tumor established from the Myc-CaP cell line [5]. Progression of this tumor model followed the clinical course of castrate resistant prostate cancer development via initial loss of AR nuclear localization to the cytoplasm before regaining AR transactivation and phenocopying the parental androgen sensitive tumor [6]. While the development of a subcutaneous castration resistant tumor model enables for rapid pre-clinical therapy studies [7], the ‘true’ clinical scenario can be still misrepresented. In efforts to not create another metastatic model through artificial means including intratibial or intracardiac injection, we undertook implantation of Myc-CaP/AS or Myc-CaP/ CR tumors orthotopically to be ‘housed’ in their natural environment.

**Figure 1:**
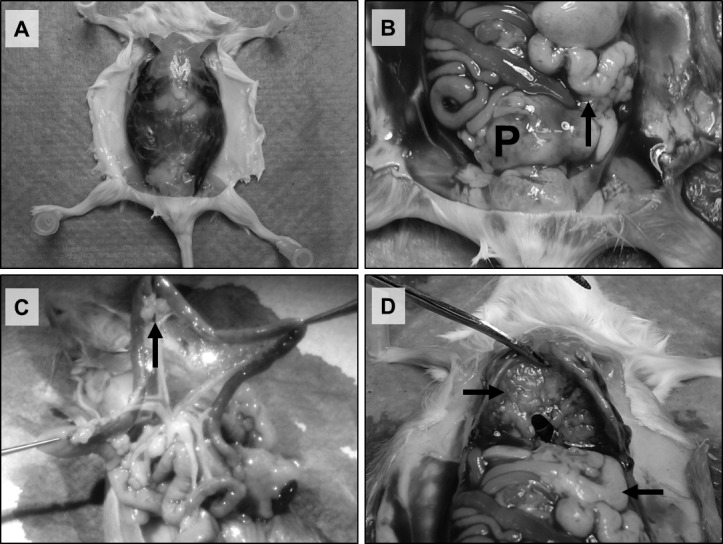
Orthotopic implant of Myc-CaP tumor pieces results in spontaneous lymph metastasis Representative pictures of a surgically castrated male wild type FVB mouse bearing a Myc-CaP/CR tumor. Macroscopic analysis displays A: Formation of abdominal ascites, B: Primary tumor (P) and adjacent lymph metastasis (arrow), C: Distal metastasis to mesenteric lymph nodes (arrow) and D: Distal metastasis to lymph tissue (lower arrow) and the diaphragm (upper arrow).

**Figure 2:**
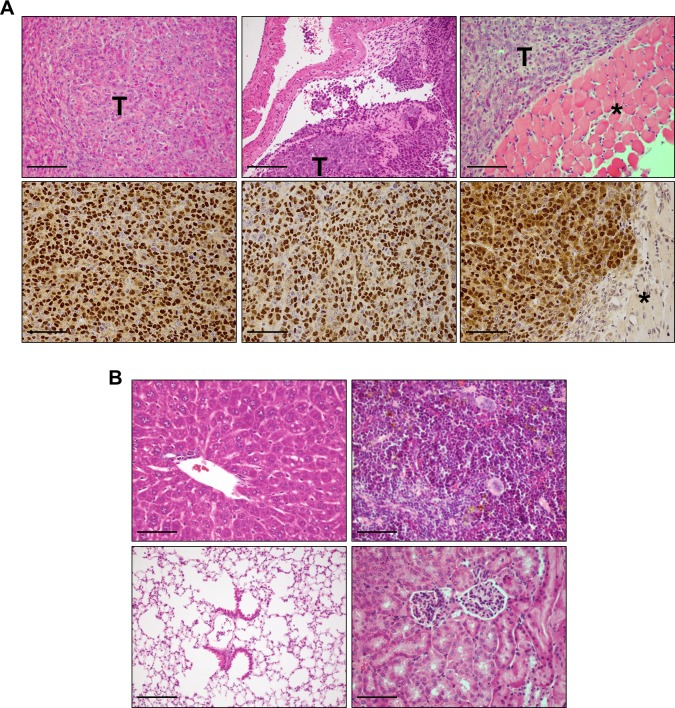
Histopathology confirmation of specific metastatic disease progression to lymph tissue A: (Upper panel) Representative H&E staining of formalin fixed paraffin embedded from Fig. 1 of primary tumor (left), lymph tissue (middle) and diaphragm (right). Tumor is indicated by (T).* indicates muscle wall of the diaphragm. (Lower panel) Corresponding IHC performed with an androgen receptor targeted antibody further confirms tumor tissue and metastatic disease. B: Representative H&E staining of liver (upper left), spleen (upper right), lung (lower left) and kidney (lower right) formalin fixed paraffin embedded tissues indicates no formation of metastatic disease to these sites. Scale bars = 500μm.

**Figure 3:**
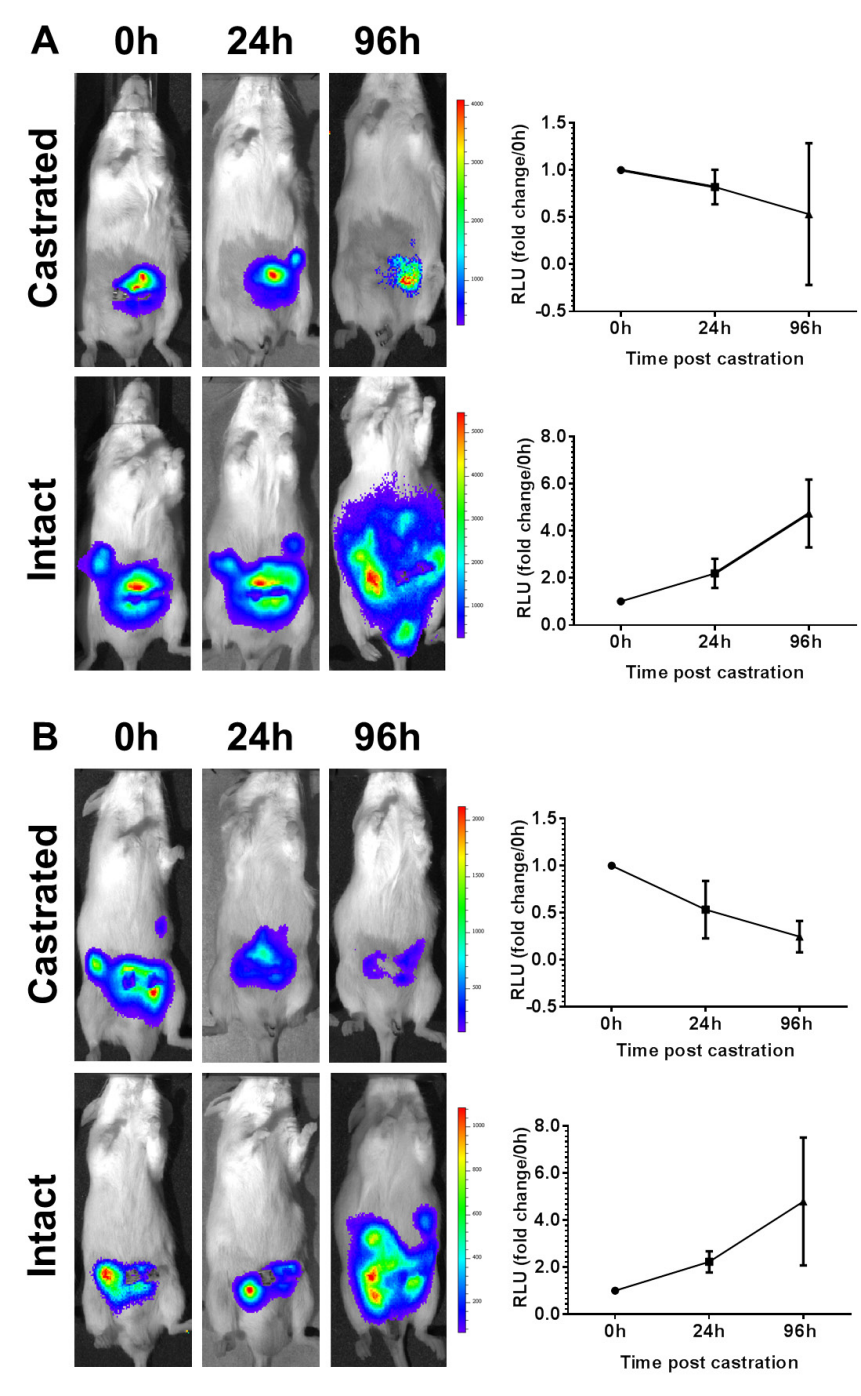
Orthotopic Myc-CaP tumors are tractable *in vivo* Intact male FVB mice bearing opthotopic Myc-CaP/AS tumors stably expressing reporter plasmids for constitutive active luciferase (Myc-CaP/AS-Luc) or androgen response elements driving luciferase (Myc-CaP/AS-ARE). Ten (10) days post implant (t = 0h) baseline biolumescence was recorded for intact and surgically castrated mice. A: Myc-CaP/AS-Luc and B: Myc-CaP/AS-ARE both indicate tumor growth and response to castration when imaged at indicated time points.

Implanted tumors to the anterior lobe of the prostate in either intact or castrated male mice were successful in establishing primary tumor growth and unexpectedly progressed to develop spontaneous metastatic disease. Upon assessment of tumor burden by necropsy and histopathology it was observed that mice bearing Myc-CaP/AS or Myc-CaP/CR tumors developed specific metastatic disease to their lymphatic tissue. Further, primary prostate cancer growth progression to metastatic disease in mice was preceded by abdominal bloating due to ascites formation, indicating possible involvement in immune/inflammatory mechanisms underlying metastatic progression (Fig. 1A). Assessment of ascites fluid from these mice did contain tumor cells (data not shown). Figure 1B-D illustrates macroscopically, development of primary Myc-CaP tumor growth with specific metastatic disease progression to lymph tissue, including pelvic, mesenteric, renal and diaphragm lymph nodes as well as connecting lymph vessels. Development of diaphragmatic metastasis was an associated morbidity in tumor bearing animals. We believe that progression of metastasis to the diaphragm maybe a result from the diaphragm lymphatics function to drain fluid from the peritoneal cavity [8]. Histopathology examination by hematoxylin and eosin (H&E) staining was performed on primary tumors and all macroscopic sites of metastatic disease (Fig. 2). Tissue samples from alternate soft tissue sites including liver, spleen, lung and kidney were also examined for micrometastasis (Fig. 3). Bone examination was excluded from this study. H&E staining revealed that liver, spleen, lung and kidney samples were negative for micrometastatic disease, rather exclusive infiltration of lymphatic tissue as indicated by Myc-CaP tumors cells with nuclear atypia were observed (Fig. 2). Further confirmation of lymph metastasis was performed by immunostaining using an antibody against the androgen receptor (Fig. 2).

Finally, we had previously shown the use of Myc-CaP/AS tumors which stably express reporter plasmids containing constitutive active luciferase (Myc-CaP/AS-Luc) or androgen response elements driving luciferase expression (Myc-CaP/AS-ARE) [7]. Mice bearing Myc-CaP/AS-Luc or Myc-CaP/AS-ARE tumors could be visually assessed by bioluminescence ten (10) days post orthotopic implantation (t=0h) (Fig. 3). Further, tumor progression in intact male mice and tumor regression in response to surgical castration was tractable using bioluminescence imaging (Fig. 3).

Overall, we demonstrate that androgen sensitive and castrate resistant Myc-CaP tumors are a powerful and viable orthotopic model when transplanted to the anterior prostate lobe of wild type male mice. Apart from primary tumor growth, these tumors progress to develop spontaneous lymph metastasis that is tractable by bioluminescence imaging. Further, the development of this *in vivo* transplant tumor model offers the possibilities to trial novel therapeutic strategies for the treatment of metastatic prostate cancer, and to investigate the process of spontaneous metastatic disease arising from its organ of origin under androgen sensitive or androgen depleted conditions in an immune competent host.

## MATERIALS AND METHODS

### Cell culture and reagents

The Myc-CaP cell line [5] was a kind gift from Dr Charles Sawyers and were cultured in DMEM medium Gibco) supplemented with 10% fetal bovine serum and 1% penicillin/streptomycin at 37°C, 5% CO_2_. Myc-CaP/ AS, Myc-CaP/CR, Myc-CaP/Luc and Myc-CaP/ARE cell lines and tumors have been described previously [7].

### *In Vivo* Animal Models

All animal studies undertaken in this study were approved by the Institute Animal Care and Use Committee (IACUC) at Roswell Park Cancer Institute. Mice were housed in an animal facility maintained on a 12-h light/ dark cycle, at a constant temperature (22±2°C) and relative humidity (55±15%). Tap water and food were available *ad libitum*. Male 4- to 6- week old FVB mice were purchased from NCI Frederick (Maryland, USA).

*Orthotopic tumor implantation*: For intraprostatic implantation, mice were anesthetized with isoflurane inhalation anesthetic. A low abdominal transverse incision was made through the skin and abdominal muscle. The bladder was grasped with forceps and pulled through the incision to expose the prostate. A tumor piece (~5mm2) was placed into the anterior lobe of prostate. The bladder was returned to the abdomen and the incision was closed using sutures. If mice received castrate resistant tumor models, surgical castration was performed within the same surgery.

### Histology/Immunohistochemistry

Mice were sacrificed by CO_2_ asphyxiation at defined time points. Tumor tissue as well as pieces of liver, kidney, spleen and lung tissue was fixed in 10% buffered formalin overnight. Lungs were also inflated with 10% buffered formalin prior to overnight fixation. All tissues were examined by routine light microscopy after hematoxylin and eosin (H&E) staining. For antigen retrieval, slides were boiled for 10 minutes in 10mM sodium citrate pH 6 solution for all antibodies. Primary antibodies were incubated at the following dilutions: Androgen Receptor (Santa Cruz N20; 1:200) was applied according cell signaling protocol. ImmPRESSTM detection system (Vector Laboratories) was used for all antibodies. Staining was visualized using 3,3′- Diaminobenzidine (DAB) (Sigma, Saint Louis, MO, FAST 3,3′-Diamino benzidine) and slides were counterstained with hematoxylin. For assessment by necropsy and histopathology in intact and castrated animals, sample size was at least 10 animals per group.

### Bioluminescence Imaging

Serial bioluminescence imaging (BLI) was carried out using the Xenogen IVIS *in vivo* Imaging System (Caliper Life Science). Animals were injected i.p. with D-luciferin potassium salt dissolved in PBS. Ten minutes after D-luciferin injection, mice were imaged under isoflurane inhalation anesthesia for detection of luciferase activity. Sample numbers for imaging experiments were: Myc-CaP/AS-Luc intact (n = 4) and castrated (n = 7) and Myc-CaP/AS-ARE intact (n = 3) and castrated (n = 4).
